# Nonconcurrent Control Use in FDA Approval of High-Risk Medical Devices

**DOI:** 10.1001/jamanetworkopen.2025.6230

**Published:** 2025-04-22

**Authors:** Maryam Mooghali, Sanket S. Dhruva, Hollin R.L. Hakimian, Vinay K. Rathi, Kushal T. Kadakia, Joseph S. Ross

**Affiliations:** 1 Department of Internal Medicine, Yale School of Medicine, New Haven, Connecticut; 2 Section of Cardiology, Department of Medicine, University of California, San Francisco School of Medicine; 3 San Francisco Veterans Affairs Health Care System, San Francisco, California; 4 Center for Outcomes Research and Evaluation, Yale-New Haven Health System, New Haven, Connecticut; 5 Department of Otolaryngology, Ohio State University Wexner Medical Center, Columbus; 6 Harvard Medical School, Boston, Massachusetts; 7 Department of Health Policy and Management, Yale School of Public Health, New Haven, Connecticut

## Abstract

**Importance:**

The US Food and Drug Administration (FDA) may consider historical controls, objective performance criteria, or performance goals as alternatives to concurrent control groups in clinical studies evaluating safety and/or effectiveness of high-risk medical devices. However, nonconcurrent control use has limitations.

**Objective:**

To examine frequency, justification, and clinically relevant details for use of nonconcurrent controls in pivotal studies supporting FDA approval of high-risk medical devices.

**Design, Setting, and Participants:**

Cross-sectional study from 2019 through 2023 of original high-risk therapeutic medical device approvals in the US.

**Main Outcomes and Measures:**

Frequency, justification, and clinically relevant details for use of historical controls, objective performance criteria, and performance goals and whether they had been established by the FDA.

**Results:**

From 2019 to 2023, the FDA approved 101 original high-risk therapeutic medical devices, of which 13 (12.9%) were not approved on the basis of any pivotal study. Among the remaining 88 approvals, 33 (37.5%) were based on pivotal studies that used concurrent controls for all analyses, 3 (3.4%) on single-group studies without the use of nonconcurrent controls, and 52 (59.1%) were based on at least 1 analysis using historical controls, objective performance criteria, or performance goals for primary safety and/or effectiveness end points. These 52 premarket approvals included 79 primary safety or effectiveness analyses using nonconcurrent controls: 9 historical controls (11.4%), 7 objective performance criteria (8.9%), and 63 performance goals (79.7%). Overall, 3 analyses (3.8%) provided justification for use of nonconcurrent controls. Clinically relevant details were provided for 19 analyses (24.1%) with respect to historical data publication dates and for 10 (12.7%) with respect to comparison of patient characteristics between pivotal studies and nonconcurrent controls. Four of 7 objective performance criteria (57.1%) and 0 of 63 performance goals had been established by the FDA.

**Conclusions and Relevance:**

This cross-sectional study of high-risk therapeutic medical device approvals by the FDA over the past 5 years found that most approvals were based on analyses using nonconcurrent controls with limited justification and clinically relevant details, potentially creating uncertainty for patients, clinicians, and payers regarding the evidence establishing device safety and effectiveness.

## Key Points

**Question:**

What are the frequency, justification, and clinically relevant details to support use of historical controls, objective performance criteria, and performance goals in studies used for US Food and Drug Administration (FDA) approval of high-risk medical devices?

**Findings:**

This cross-sectional study of high-risk therapeutic medical device approvals from 2019 through 2023 found that 51% were based on primary safety or effectiveness analyses that used nonconcurrent, as opposed to concurrent, controls. FDA documentation provided limited information about justification and clinically relevant details to support use of these nonconcurrent controls.

**Meaning:**

Nonconcurrent controls are used frequently for FDA approval of high-risk medical devices, potentially limiting safety and effectiveness assessments.

## Teaser

This cross-sectional study evaluates the frequency, justification, and clinically relevant details for use of nonconcurrent controls in pivotal studies supporting US Food and Drug Administration approval of high-risk medical devices.

## Introduction

The US Food and Drug Administration (FDA) classifies medical devices by risk, which has implications for the amount of information manufacturers must submit for market authorization.^1^ High-risk medical devices “sustain or support life, are implanted, or present potential unreasonable risk of illness or injury” and generally undergo review through the premarket approval (PMA) pathway,^1^ requiring evidence from clinical studies demonstrating reasonable assurance of safety and effectiveness.^1^ Typically, clinical research studies use concurrent control groups, such as placebo plus standard of care, to differentiate an intervention’s impact on patients from effects influenced by other factors, such as quality of care.^2^ However, for therapeutic medical devices and related procedures, placebo assignments (often referred to as sham controls) are more difficult to design.^3,4^ Accordingly, in certain circumstances, FDA guidance suggests that use of nonconcurrent controls may be appropriate, such as those involving diseases with high and predictable mortality or signs and symptoms of predictable duration or severity.^3,5,6^

The 3 most common nonconcurrent controls are historical controls, objective performance criteria, and performance goals (Box). Historical controls refer to data collected from prior studies of patients with the disease of interest compared with results experienced by patients in a contemporary clinical trial.^5,6^ Objective performance criteria are numerical target values derived from previous clinical trials or registries that are frequently used in binary (pass or fail) determinations,^5,6^ such as a successful arrhythmia conversion rate of 90% after implantable cardioverter-defibrillator placement. Objective performance criteria are ideally constructed based on patient-level meta-analyses.^5,7,8^ However, many device technologies are less established and there are few validated objective performance criteria.^5,8^ Consequently, the FDA frequently relies on performance goals, which represent numerical values (point estimates) deemed acceptable for assessing device safety and effectiveness, often based on the upper or lower confidence limit of a safety and/or effectiveness end point,^5,6^ such as a major adverse event-free rate of more than 85% after cochlear implant surgery (based on the 95% CI lower bound). The underlying data used for establishing performance goals are generally less robust compared with those used for objective performance criteria.^5,6,8^

Box. **Definition of Historical Controls, Objective Performance Criteria, and Performance Goals in Clinical Studies^a^**

**Historical controls:** The historical data from previous studies that are quantitatively compared with the results of a contemporary clinical trial.
**Objective performance criteria:** Numerical target values, derived from previous clinical trials or registries, that may be used in a binary (pass/fail) approach for safety or effectiveness outcomes.
**Performance goals:** Numerical values (point estimates) deemed acceptable for assessing the safety and effectiveness of investigational devices, which might be based on the upper or lower confidence limit of a safety and/or effectiveness end point. As the data used to generate performance goals are not as robust as for objective performance criteria, the level of evidence is inferior.

^a^
Sources: US Food and Drug Administration. Design Considerations for Pivotal Clinical Investigations for Medical Devices Guidance Document^5^ and US Food and Drug Administration. Use of Real-World Evidence to Support Regulatory Decision-Making for Medical Devices Guidance Document.^6^

Historical controls, objective performance criteria, or performance goals are most useful when it is difficult to conduct studies with conventional control groups (eg, rare conditions or indications lacking clinical equipoise for a concurrent control).^5^ However, nonconcurrent control data may have limitations, such as lack of generalizability to contemporary patient populations receiving standard of care, which can bias results and produce inaccurate effect estimates.^9,10^ Most high-risk cardiovascular devices approved by the FDA from 2000 to 2011 were not compared with concurrent controls, but instead were often evaluated relative to nonconcurrent controls or no control.^11^ Notably, fewer than 30% of objective performance criteria and performance goals used as comparators for these cardiovascular devices had been previously established by FDA.^11^

FDA and Congress have recently emphasized the use of real-world evidence, defined as clinical evidence regarding the usage and potential benefits or risks of a medical product derived from analysis of real-world data.^6,12^ These data relate to patient health status and/or the delivery of health care and are derived from routinely collected sources and include use of nonconcurrent controls in studies informing FDA approval. We sought to build upon prior research^11^ to inform these ongoing efforts and examined all high-risk therapeutic medical devices over the past 5 years and identified the frequency with which nonconcurrent controls were used to support FDA approval, along with justifications and clinically relevant details to support their use.

## Methods

In accordance with 45 CFR §46, this cross-sectional study was exempt from ethics review and informed consent because it used public, nonidentifiable data and was not human participant research. We followed the Strengthening the Reporting of Observational Studies in Epidemiology (STROBE) reporting guideline.^13^ Data were collected by 1 investigator (M.M. or H.R.L.H.). To reduce bias, a random 20% of collected data were validated by the other investigator, with any disagreements resolved by consensus.

### Study Sample

Using the Devices@FDA database,^14^ we identified all high-risk medical devices that received FDA original premarket approval from January 1, 2019, to December 31, 2023, excluding devices with diagnostic indications. For each device, we reviewed the FDA’s Summary of Safety and Effectiveness Data (SSED) to determine whether the pivotal clinical study or studies informing FDA approval relied on analyses based on historical controls, objective performance criteria, or performance goals for primary safety and/or effectiveness end point(s). Additionally, we identified instances where the pivotal clinical study’s primary end point was initially compared with a concurrent control and not met, but the device was subsequently approved based on comparison(s) with nonconcurrent controls. We excluded medical devices without pivotal studies supporting approvals, including those relying on a literature review and/or previous FDA authorizations (such as devices reclassified through the 515 Program Initiative^15,16^).

### Device Characteristics

Using previously described methods,^17^ we extracted the following FDA-designated device characteristics from the Devices@FDA database: implantable status, life-sustaining and/or supporting status, Breakthrough Devices Program designation, and therapeutic area. To examine the extent to which device types have been established, as a proxy for feasibility of developing alternatives to concurrent controls, we sought to quantify the presence of follow-on technologies within a given device product type. To do so, we identified the date when the corresponding product code(s) of the approved device first became active (ie, the date when the first device with the same product code had been approved) and calculated the duration between approval of the first device with the same product code and the new device’s premarket approval based on nonconcurrent controls. We also estimated technological relevance of nonconcurrent control data by determining the number of original and supplemental premarket approvals for devices with the same product code during that time frame (ie, between approval of the first device with the same product code and the new device’s premarket approval).^18^

### Pivotal Study Characteristics

For each pivotal study mentioned in the SSEDs, we extracted the National Clinical Trial number.^18^ We then reviewed SSEDs and ClinicalTrials.gov records to extract information about pivotal study type (clinical trial vs registry-based, using the Agency for Healthcare Research and Quality definition^19^), number of groups , and number of patients.

### Use of Historical Controls, Objective Performance Criteria, or Performance Goals

For each analysis that compared end point(s) with historical control, objective performance criterion, or performance goal, we determined whether the end points were met. Next, we abstracted any justification explicitly stated in SSEDs for the use of nonconcurrent controls (ie, why a concurrent comparator was not included), including whether the disease treated met the FDA’s established criteria for nonconcurrent control use (ie, diseases with high and predictable mortality or signs and symptoms of predictable duration or severity^5^). We used SSEDs to determine whether objective performance criteria or performance goals had been established by the FDA before the device’s approval (as opposed to developed by the sponsor), and whether they were based on previous clinical studies or published literature. Moreover, we determined whether historical controls, objective performance criteria, or performance goals had been prespecified on ClinicalTrials.gov registration entries.

We next sought to assess clinically relevant details to support the use of nonconcurrent controls. To do so, we determined whether SSEDs included comparisons between historical control data or those used to establish objective performance criteria or performance goals with the study data for the newly approved device in terms of patient population characteristics (sex, age, race, ethnicity, disease stage or severity, and comorbidities). Additionally, we identified the date when historical control data or data used to establish objective performance criteria or performance goals were published and then calculated the duration to the device approval date.

### Statistical Analysis

We used descriptive statistics to characterize the sample. We used Excel 2018 (Microsoft) to record and summarize data.

## Results

### Study Sample

From 2019 to 2023, the FDA approved 161 FDA original high-risk medical devices, 56 of which (34.8%) were diagnostic and excluded. Among the remaining 105 therapeutic devices, 4 (3.8%) lacked a publicly available SSED, and 13 (12.4%) were not approved on the basis of any pivotal study (Figure).

**Figure.**
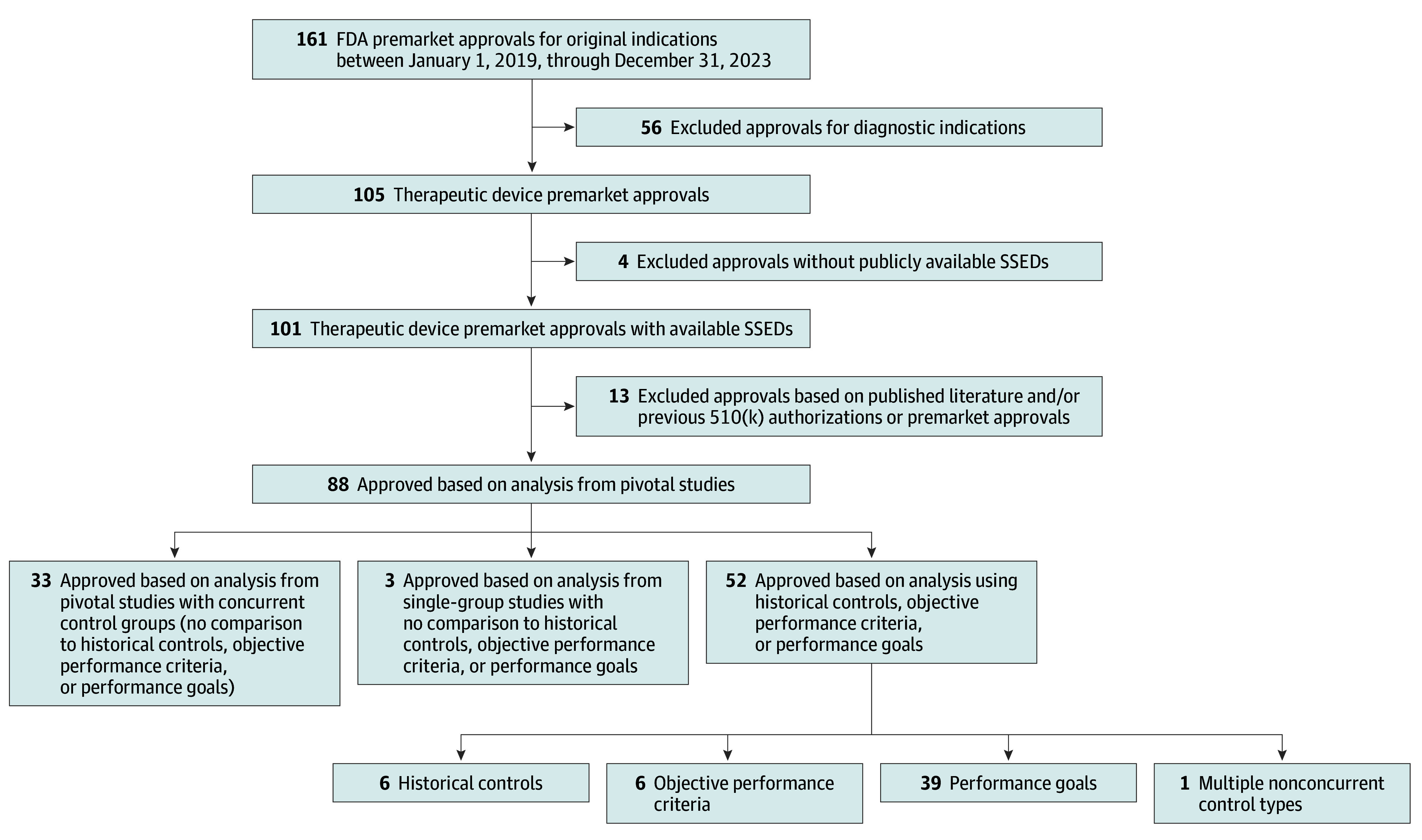
Construction of Study Sample of Therapeutic Medical Devices With Original Premarket Approval Based on Historical Controls, Objective Performance Criteria, or Performance Goals FDA indicates US Food and Drug Administration; SSED, Summary of Safety and Effectiveness Data.

Of the remaining 88 high-risk devices, FDA approval for 33 (37.5%) was based on pivotal studies that based all comparisons on concurrent controls for all analyses, 3 (3.4%) on single-group studies without the use of nonconcurrent controls, and 52 (59.1%) on at least 1 primary safety or effectiveness analysis using historical controls, objective performance criteria, or performance goals.

### Device Characteristics

Among the 52 devices approved based on analyses using nonconcurrent controls, 21 (40.4%) were life-sustaining/supporting, 39 (75.0%) were implantable, 10 (19.2%) were breakthrough-designated, and 33 (63.5%) were used to treat cardiovascular indications (Table 1). The median (IQR) duration between approval of the first device with the same product code and approval of the newly approved device was 5.2 (0-20.8) years, during which time there were a median (IQR) of 1 (0-8) original and 10 (0-208) supplemental premarket approvals for devices with the same product code.

**Table 1.** Characteristics of the US Food and Drug Administration’s Original Premarket Approval for Therapeutic Medical Devices Based on Historical Controls, Performance Goals, or Objective Performance Criteria, 2019 to 2023

Device characteristic	Devices, No. (%) (N = 52)
Life-sustaining	21 (40.4)
Implantable	39 (75.0)
Breakthrough devices program-designated	10 (19.2)
Therapeutic area	
Cardiovascular	33 (63.5)
Orthopedic	4 (7.7)
Gastroenterology/urology	3 (5.8)
Neurology	3 (5.8)
Ophthalmic	3 (5.8)
Ear, nose, and throat	2 (3.8)
Obstetrics/gynecology	2 (3.8)
Radiology	1 (1.9)
Anesthesiology	1 (1.9)
Timelines and No. of approvals for similar premarket approval devices	
Duration between approval of the first device with the same product code and current approval date, median (IQR), y	5.2 (0-20.8)
No. of original premarket approvals for devices with the same product code during this time frame, median (IQR)	1 (0-8)
No. of supplemental premarket approvals for devices with the same product code during this time frame, median (IQR)	10 (0-208)

### Pivotal Study Characteristics

Of the 52 premarket approvals using nonconcurrent controls, 47 (90.4%) were based on clinical trials and 5 (9.6%) on registry-based studies, with a median (IQR) of 191 (141-246) patients in the investigational device group. Overall, 42 device approvals (80.8%) using nonconcurrent controls were based on single-group studies, and 10 (19.2%) were based on studies that included comparator groups. For 7 of the 10 studies (70.0%) with comparator groups, the concurrent comparator was used for primary effectiveness analysis, while the nonconcurrent control was used for primary safety analysis.

### Use of Historical Controls, Objective Performance Criteria, or Performance Goals

Among the 52 premarket approvals using nonconcurrent controls, 6 (12.8%) were based on analyses using historical controls, 6 (12.8%) on objective performance criteria, and 39 (83.0%) on performance goals; 1 premarket approval (2.1%) used an objective performance criterion for establishing effectiveness and a performance goal for establishing safety (Table 2). The use of these nonconcurrent control study designs was prespecified on ClinicalTrials.gov for 11 of the 52 approvals (21.2%): 1 historical control approval, 2 objective performance criteria approvals, and 8 performance goals approvals.

**Table 2.** Evidence Informing the US Food and Drug Administration’s Original Premarket Approvals for Therapeutic Medical Devices Based on Historical Controls, Performance Goals, or Objective Performance Criteria, 2019 to 2023

Evidence informing premarket approval	Devices, No. (%) (N = 52)
Historical control	
Overall	6 (12.8)
For safety only	1 (16.7)
For effectiveness only	1 (16.7)
For separate safety and effectiveness analyses	3 (50.0)
For a single analysis establishing both safety and effectiveness	1 (16.7)
Objective performance criterion	
Overall	6 (12.8)
For safety only	2 (33.3)
For effectiveness only	4 (66.7)
Performance goal	
Overall	39 (83.0)
For safety only	6 (15.4)
For effectiveness only	5 (12.8)
For separate safety and effectiveness analyses	23 (59.0)
For a single analysis establishing both safety and effectiveness	5 (12.8)
Mixed comparators	
Overall	1 (2.1)
Objective performance criterion for effectiveness, performance goal for safety	1 (100.0)

#### Historical Control

Among the 6 premarket approvals based on analyses using historical controls, 3 (50.0%) reported separate safety and effectiveness analyses, 1 (16.7%) a single analysis establishing both safety and effectiveness, 1 (16.7%) a safety analysis only, and 1 (16.7%) an effectiveness analysis only.

In total, 9 analyses used historical controls to establish device safety and effectiveness (4 [44.4%] safety, 4 [44.4%] effectiveness, and 1 [11.1%] establishing both safety and effectiveness) (Table 3). The primary end points were met for all 9 analyses (100.0%). For 2 of the 9 analyses (22.2%), SSEDs documented justification for using historical controls. The median (IQR) duration from publication of historical control data to the new device approval for these 9 analyses was 11.4 (6.5-13.1) years.

**Table 3.** Use of Historical Controls, Objective Performance Criteria, or Performance Goals in the US Food and Drug Administration’s (FDA) Analysis for Original Premarket Approvals for Therapeutic Medical Devices, 2019 to 2023 (N = 79)

Criteria	Approvals, No. (%)		
	HC (n = 9)	OPC (n = 7)	PG (n = 63)
Primary end point of safety or effectiveness analysis was met	9 (100.0)	7 (100.0)	62 (98.4)
SSEDs provided justification for the use of HC, OPC, or PG^a^	2 (22.2)	0	1 (1.6)
SSEDs provided publication dates of historical data for HC, OPC, or PG	9 (100.0)	2 (28.6)	8 (12.7)
SSEDs compared historical data for HC, OPC, or PG with pivotal study data^b^	6 (66.7)	1 (14.3)	3 (4.8)
SSEDs provided origin of OPC or PG^c^	NA	6 (85.7)	52 (82.5)
OPC or PG had been established by FDA^c^	NA	4 (57.1)	0
OPC or PG was based on published literature or previous trials^c^	NA	5 (71.4)	52 (82.5)

Abbreviations: HC, historical control; NA, not applicable; OPC, objective performance criterion; PG, performance goal; SSED, Summary of Safety and Effectiveness Data.

^a^ SSEDs provided explanations regarding why studies with conventional control groups were not conducted.

^b^ SSEDs compared historical data for HC, OPC, or PG with pivotal study data in terms of the study designs and/or population characteristics..

^c^ These categories are not mutually exclusive.

#### Objective Performance Criteria

Among the 7 premarket approvals based on analyses using objective performance criteria, 2 (28.6%) reported safety analyses only, and 5 (71.4%) reported effectiveness analyses only; the primary end points were met for all 7 (100.0%). No SSEDs included justification for the use of objective performance criteria. Additionally, 2 approvals (28.6%) had SSEDs that stated the publication dates of data used to establish the objective performance criteria used for the analyses; the duration from publication of evidence supporting establishing objective performance criteria data to the new device approval were 4.4 and 8.0 years.

SSEDs documented that 4 of the 7 objective performance criteria (57.1%) (nonmutually exclusive) had been previously established by the FDA and 5 (71.4%) were based on previous trials. The source of objective performance criterion for 1 approval (14.3%) was not provided within the SSED.

#### Performance Goals

Among the 40 premarket approvals based on analyses using performance goals, 23 (57.5%) used performance goals for separate safety and effectiveness analyses, 5 (12.5%) used a single analysis establishing both safety and effectiveness, 7 (17.5%) used safety analyses only, and 5 (12.5%) used effectiveness analyses only. In total, 63 analyses used performance goals to establish device safety and effectiveness (30 safety analyses, 28 effectiveness analyses, and 5 analyses establishing both safety and effectiveness).

Among these 63 analyses, The primary end point was met for 62 analyses (98.4%). For 1 of the 63 analyses (1.6%), SSED documented justification for using performance goals: since the primary end point against concurrent comparator was not met, a comparison to a performance goal was conducted.^20^ The SSEDs for the remaining 62 analyses (98.4%) lacked justification. Publication dates of data used to establish performance goals were provided in the SSEDs for 8 of the 63 analyses (12.7%). Among these, the median (IQR) duration from publication of evidence supporting establishing the performance goal to the approval was 6.8 (5.0-8.8) years.

SSEDs documented that the performance goals used for 52 of the 63 analyses (82.5%) were based on published literature or previous trials; the origin of the remaining 11 (17.5%) was not stated. No performance goals had been previously established by FDA.

#### Representativeness of Historical Data

Among all 79 analyses using nonconcurrent controls for the 52 premarket approvals, 10 (12.7%) included a comparison of patient characteristics between those receiving the therapeutic device in pivotal studies and nonconcurrent controls: 6 of 9 historical controls (66.7%), 1 of 7 objective performance criteria (14.3%), and 3 of 63 performance goals (4.8%). The population characteristics were comparable for all 10 of these analyses (100.0%).

## Discussion

In this study of high-risk therapeutic medical devices approved by the FDA over the past 5 years, we found that over half of approvals relied on primary safety or effectiveness analyses of pivotal studies using historical controls, objective performance criteria, or performance goals, as opposed to studies with concurrent controls. Publicly available regulatory documents for almost all of these approvals lacked justification for the use of nonconcurrent controls, and only 12.7% compared the characteristics of the nonconcurrent control patients with those enrolled in the pivotal studies. While the FDA is required by law to follow the least burdensome provision and strives to balance scientific certainty with technological innovation,^21^ frequent use of nonconcurrent controls, particularly without justification and clinically relevant details, may create uncertainty for patients, clinicians, and payers regarding the safety and effectiveness of FDA-approved high-risk medical devices.

Nonconcurrent controls may be ethically and scientifically justified for diseases with high and predictable mortality (eg, coronary artery perforation) or signs and symptoms of predictable duration or severity (eg, severe aortic stenosis among patients at prohibitive surgical risk).^5^ However, studies that use nonconcurrent controls could be prone to selective reporting of favorable outcomes, and their nonrandomized designs increase the risk of confounding when comparing new and historical data, raising ethical concerns about patient harm.^10,22,23^ These are particularly important given differences in patient populations, changes in standard of care over time, and variations in data collection methods.^9^ Furthermore, comparisons using objective performance criteria or performance goals cannot establish intervention superiority or noninferiority, limiting inferences about new devices’ safety and effectiveness.^24^

In addition to their inherent limitations, the lack of publicly available information about the nonconcurrent control evidence informing approvals underscores concerns about data quality.^11,25,26^ Timelines for establishing objective performance criteria and performance goals were infrequently available in FDA approval documents, and when they were, data were over 7 years old. Historical control populations were even older, at more than 11 years. Such outdated information may not accurately reflect contemporary clinical and technological standards of care, especially given rapid updates to and adoption of medical device technologies.^27,28^ Additionally, few premarket approval SSEDs reported comparisons of the historical data patients to those of the pivotal studies, leading to uncertainty regarding whether the populations are sufficiently similar.

Most high-risk cardiovascular devices subject to recall under the FDA’s most serious safety designation were not compared with an active control during premarket approval, suggesting that use of nonconcurrent controls in clinical studies to support market authorization may pose safety risks to patients.^29^ For example, the VICI VENOUS STENT System, intended for the treatment of symptomatic venous outflow obstruction and approved based on performance goals, was issued a class 1 recall nearly 3 years after approval due to reports of stent migration following implantation, affecting over 30 000 devices.^30^ The FDA could consider using nonconcurrent controls only in situations where conducting clinical trials with concurrent controls is unfeasible or unethical, and when used, should only rely on the most up to date performance goals and objective performance criteria.^6^ Additionally, clear justification and evidence on the predictability of disease mortality and morbidity could be reported in the SSED. Furthermore, the FDA could require sponsors to publicly disclose the clinically relevant details, such as historical control data collection dates and patient demographic and clinical characteristics, in addition to including standardized comparisons between the pivotal study and nonconcurrent control patients.

Eighty percent of premarket device approvals using nonconcurrent controls relied on performance goals, which are generally less robust than objective performance criteria,^5,6^ and none had been previously established by the FDA. Given that for many of these approvals, devices with the same product code have been available on the market for over half a decade, there may be opportunities to establish validated objective performance criteria, ideally before pivotal study initiation. Establishing these criteria requires joint efforts between the FDA, sponsors, patients, medical or scientific societies, and other stakeholders.^31^ Additionally, the FDA could mandate postmarketing studies for approvals based on nonconcurrent control studies to continue evaluating device safety and effectiveness in real-world settings. These data could inform validation of nonconcurrent controls, ensuring that they remain relevant and reflective of current clinical practice.^6,8^

### Limitations

There are study limitations to consider. First, we only relied on publicly available data, including the information in SSEDs. While justification or clinically relevant details about the use of nonconcurrent controls may not always be included in SSEDs, SSEDs are required to provide detailed premarket approval information summaries, including the clinical evidence and the FDA’s analyses of the evidence that served as the basis for the FDA’s approval decisions.^32^ Second, we only examined original indication approvals, while nonconcurrent controls may also be used for supplemental indication approvals. Therefore, our study may underestimate the role of nonconcurrent controls in regulatory decision-making for high-risk therapeutic devices.

## Conclusions

In this cross-sectional study of high-risk therapeutic medical device premarket approvals between 2019 to 2023, we found that over half of original indication approvals relied on analyses of pivotal studies using historical controls, objective performance criteria, or performance goals to establish device safety or effectiveness. There was limited information about justification and clinically relevant details to support use of these nonconcurrent controls, and few had been previously established by the FDA, which could negatively impact clinical, regulatory, and coverage decisions. Policy reforms promoting greater transparency would help patients, clinicians, and payers to better understand the evidence used to establish high-risk medical device safety and effectiveness.

## Notes

Supplement.
Data Sharing Statement
