# Supplementary material for: Nonconcurrent Control Use in FDA Approval of High-Risk Medical Devices
Source: JAMA Netw Open. 2025 Apr 22;8(4):e256230. doi: 10.1001/jamanetworkopen.2025.6230 (PMC12015671; doi:10.1001/jamanetworkopen.2025.6230)
Supplement: Supplement. — Data Sharing Statement [file jamanetwopen-e256230-s001.pdf]

Data Sharing Statement

Mooghali. Nonconcurrent Controls in FDA Approval of High-Risk Medical Devices. *JAMA Netw Open*. Published April 22, 2025. doi:10.1001/jamanetworkopen.2025.6230

Data
Data available: No

Additional Information
Explanation for why data not available: Relevant data are available on reasonable request from the corresponding author.
